# Targeted Selected Reaction Monitoring Mass Spectrometric Immunoassay for Insulin-like Growth Factor 1

**DOI:** 10.1371/journal.pone.0081125

**Published:** 2013-11-21

**Authors:** Eric E. Niederkofler, David A. Phillips, Bryan Krastins, Vathany Kulasingam, Urban A. Kiernan, Kemmons A. Tubbs, Scott M. Peterman, Amol Prakash, Eleftherios P. Diamandis, Mary F. Lopez, Dobrin Nedelkov

**Affiliations:** 1 Thermo Fisher Scientific, Tempe, Arizona, United States of America; 2 Thermo Fisher Scientific, BRIMS, Cambridge, Massachusetts, United States of America; 3 University Health Network and University of Toronto, Toronto, Ontario, United States of America; Moffitt Cancer Center, United States of America

## Abstract

Insulin-like growth factor 1 (IGF1) is an important biomarker of human growth disorders that is routinely analyzed in clinical laboratories. Mass spectrometry-based workflows offer a viable alternative to standard IGF1 immunoassays, which utilize various pre-analytical preparation strategies. In this work we developed an assay that incorporates a novel sample preparation method for dissociating IGF1 from its binding proteins. The workflow also includes an immunoaffinity step using antibody-derivatized pipette tips, followed by elution, trypsin digestion, and LC-MS/MS separation and detection of the signature peptides in a selected reaction monitoring (SRM) mode. The resulting quantitative mass spectrometric immunoassay (MSIA) exhibited good linearity in the range of 1 to 1,500 ng/mL IGF1, intra- and inter-assay precision with CVs of less than 10%, and lowest limits of detection of 1 ng/mL. The linearity and recovery characteristics of the assay were also established, and the new method compared to a commercially available immunoassay using a large cohort of human serum samples. The IGF1 SRM MSIA is well suited for use in clinical laboratories.

## Introduction

Insulin-like growth factor 1 (IGF1) is an important biological protein implicated in many physiological process - from cell proliferation, differentiation and apoptosis, to tissue growth and organ specific functions [1]. IGF1 has been traditionally assayed from human plasma or serum samples using enzymatic or radioimmunoassays, but not without some issues – primarily associated with the dissociation from the IGF-binding proteins (IGFBPs) [2], and the dynamic range of the assays [3], [4]. The proliferation of mass spectrometry (MS) in the late 90's prompted several efforts toward its implementation in IGF1 detection. In 2001, two groups reported on the development of HPLC electrospray mass spectrometry methods for quantification of IGF1 from standard samples [5], [6]. In 2004 Nelson *et al*., described an immunoaffinity-based MALDI-TOF MS method for quantification of IGF1 from human plasma samples [7]. A few years later, Kirsch *et al*., developed an IGF1 quantification method using liquid chromatography coupled to isotope dilution mass spectrometry [8]. Several more MS-based methods were published in 2008 and 2009, some of which included IGF1 immunoaffinity retrieval [9], or acetonitrile plasma depletion step in front of the LC-MS/MS [10]. The last few years have seen the convergence of the IGF1 analytical methods toward solid-phase extraction sample preparation and LC-MS [11]–[15].

The solid phase extraction method was developed for the first IGF1 immunoassays in the 80s and 90s [16]–[18]. The method, which involves acidic disruption of the IGF1-IGFBP complex and ethanol precipitation of the IGFBPs - leaving free IGF1 in solution, has changed little since then, and most MS-based workflows have incorporated it without any significant modifications. However, it seems that the high-end capabilities of MS could be better matched to a simpler, high-throughput IGF1 sample preparation method. In this work we have adopted and modified the sample preparation approach described by Nelson et al [7], and combined it with a mass spectrometric immunoassay (MSIA) [19], [20] method using automated LC-MS/MS for selected reaction monitoring (SRM) and long R3 IGF1 (LR3-IGF1) as an internal reference standard. The resulting IGF1 SRM MSIA was characterized, validated, and employed in screening of clinical human plasma samples, and the data compared to that obtained with a clinical immuno analyzer.

## Materials and Methods

### Reagents

Polyclonal rabbit anti-human IGF1 affinity purified antibody (Cat. No. PA0362), recombinant human IGF1 (Cat. No. CRI500c), and recombinant human LR3-IGF1 (Cat. No. LRM001) were obtained from Cell Sciences (Canton, MA). Custom MSIA Pipette Tips (Cat. No. 991CUS02), and phosphate buffered saline (PBS, 28374) were obtained from Thermo Fisher Scientific (San Diego, CA). Ammonium bicarbonate (Cat. No. 09830), calcium chloride (C1016), dithiothreitol (43815), glucagon (G2044), iodoacetamide (I1149), and TWEEN 20 (P7949) were obtained from Sigma-Aldrich (St. Lous, MO). Bovine serum albumin (BSA, Cat. No. 126609), and formic acid (11670) were obtained from EMD Millipore (Billerica, MA). Sterile water (Cat. No. AB02120), acetonitrile (AB00120), isopropyl alcohol (AB00866), SDS (AB01920), and trifluoroacetic acid (TFA, AB02010) were purchased from American Bioanalytical (Natick, MA). Sequencing grade modified trypsin (Cat. No. V511) was obtained from Promega (Madison, WI).

### Human serum and plasma samples

For the method development and validation, several human plasma and serum samples were purchased from ProMedDX (Norton, MA, USA). The samples were received labeled only with a barcode and supplied with an accompanying specification sheet containing information about the gender and age of the donor. For the method comparison studies, 289 serum samples were obtained from the University Health Network (Toronto, ON, Canada), under the approval of the University Health Network Research Ethics Board (REB # 09-0486-T) for the research use of left over routine clinical specimens in the Core Diagnostic Laboratory. Written informed consent from the participants was not required based on the Tri-Council Policy Statement: Ethical Conduct for Research Involving Humans (TCPS2, Article 3.7). The samples were received numbered and without any identifiers, and with their IGF1 levels determined using the Siemens Immulite 2000 IGF-1 assay. All samples were aliquoted and stored at −80°C until used.

### Preparation of standards and analytical samples

For the standard curve generation, the IGF1 stock (1 g/L) was serially diluted to 1,500; 1,000; 500; 100; 25; 10; 5; and 1 ng/mL, with 10 mM PBS containing 1% (w/v) BSA (standards buffer). The internal reference standard (LR3-IGF1, 1 g/L) was also serially diluted in standards buffer to a final concentration of 500 ng/mL. The analytical samples were prepared in individual wells of a microtiter plate by combining 100 µL sample buffer (10 mM PBS w/0.3% (w/v) SDS), 20 µL of the 500 ng/mL LR3-IGF1 solution, and 40 µL of either the IGF1standards (for the standard curve generation) or undiluted human plasma or serum. The microplates were then shaken at room temperature for 1 h on an orbital shaker, at 1,000 rpm, to dissociate the IGF1 from the IGFBPs.

### Mass spectrometric immunoassay

The immunoaffinity retrieval of IGF1 and LR3-IGF1 from samples was performed using MSIA-Tips derivatized with the IGF1 antibody, which were prepared as previously described [21]. The MSIA-Tips were mounted onto the head of the Versette Automated Liquid Handler (Thermo Fisher Scientific, Hudson, NH) and initially rinsed with assay buffer (10 mM PBS w/0.1% TWEEN 20), with 10 cycles (1 cycle consisting of a single aspiration and dispense of a 100 µL volume, ∼3 s), from a single 150 µL buffer aliquot placed in the well of a microplate. Next, the MSIA-Tips were immersed into the wells of the microplate containing the samples, and 100 aspirations and dispense cycles were performed (100 µL volumes each), allowing for simultaneous affinity capture of IGF1 and LR3-IGF1. The MSIA-Tips were then rinsed with assay buffer (100 cycles) from another microplate, and twice with water (10 cycles each) from two more microplates (100 µL volumes aspiration and dispenses, from 150 µL placed in each well). The captured proteins were eluted with 33% acetonitrile/0.4% (v/v) TFA by aspirating and dispensing a 20 µL volume 30 times, from a total of 50 µL in the wells of a microplate. The proteins-containing eluates were dried down in a SpeedVac concentrator until dry, and re-suspended in 30 µL 30% N-propanol/100 mM ammonium bicarbonate pH 8.5/10 mM DTT. The microplate was sealed and vortexed for 30 s, spun down, and incubated at 37°C for 30 min to reduce disulfides. A 2.4 µL aliquot of 0.5 M iodoacetamide (in 100 mM ammonium bicarbonate, pH 8.5) was added into each well, and the microplate was sealed again, vortexed for 30 s, spun down, and incubated in the dark at room temperature for 30 min. A 92.5 µL aliquot of 5 mM CaCl_2_/0.1 M ammonium bicarbonate pH 8 warmed to 50°C was added to each well, and digestion of the reduced and alkylated proteins was initiated by adding 25 µL of 4 mg/L warm (50°C) trypsin to each well. The plate was sealed, vortexed for 60 s, spun down and incubated at 50°C for 2 h. The plate was then cooled down at 4°C for 5 min, and 2.3 µL of glucagon (1 g/L in 0.2% formic acid; as a peptide carrier) and 3 µL of 100% formic acid were added to each well. The microplate was positioned in an auto sampler (PAL HTC Accela1-TMO, Thermo Scientific) and the entirety of each sample (155 µL) was injected onto a 2.1-mm×100-mm Accucore aQ 2.6-µm particle C18 column (Thermo Fisher Scientific). Reversed-phase separations were carried out on an Accela 1250 pump (Thermo Fisher Scientific) at a flow rate of 240 µL/min. Solvent A was 0.2% formic acid in LC-MS grade water, and solvent B was 0.2% formic acid in LC-MS grade acetonitrile (Optima grade reagents, Thermo Fisher Scientific). SRM assays were developed on a TSQ Vantage triple quadrupole mass spectrometer equipped with a HESI-II source (Thermo Fisher Scientific). A mass window of full-width at half maximum of 0.7 (unit resolution) was used in the SRM assays, as a result of the immuno-enriched samples having high signal-to-noise. Pinpoint software (Thermo Fisher Scientific) was used for targeted protein quantification. SRM transitions from the simultaneously immuno-enriched and digested IGF1 (residues 1–21) and LR3-IGF1 (residues 17–34) were monitored, with the peak area ratio of IGF1 to LR3-IGF1 used for quantification.

## Results and Discussion

### Sample preparation

The most common IGF1 sample preparation protocol utilizes acidic conditions to dissociate the IGF1-IGFBPs complex, and ethanol to precipitate IGFBPs from the sample, leaving IGF1 free in solution for subsequent analysis [16]–[18]. An alternative approach is to release the IGF1 from its complex, and assay the protein directly, without further sample manipulation which might result in some IGF1 loss. Nelson *et al*., [7] devised an IGF1 sample preparation method that utilized sodium dodecyl sulphate (SDS) to dissociate the IGF1 from the complex with the IGFBPs. We adopted the SDS-based method in the current work, but with a few modifications. First, the optimal SDS concentration at which all of the IGF1 is dissociated from the IGFBPs was re-examined and determined empirically. Next, the SDS concentration was optimized so that it does not interfere with the antibody-antigen interaction (i.e., does not denature the protein or the antibody). This was particularly important because, to avoid any re-association of IGF1 with the IGFBPs, we eliminated any addition of extra assay buffer to the analytical samples (which was part of the method described by Nelson et al., [7]). The final protocol consisted of adding IGF1 sample and LR3-IGF1 standard aliquots to a microplate well containing PBS sample buffer with 0.3% SDS, and shaking the plate for 60 min at room temperature, after which the IGF1 and LR3-IGF1 were immunoaffinity retrieved from the samples. In this sample preparation method the transfer of reagents is minimized, and the IGF1 sample never leaves the microplate, thus ensuring more accurate IGF1 measurement.

### Internal Reference Standard

Another key feature of the sample preparation is addition of the internal reference standard (IRS) at the beginning of the assay. It is important that the IRS goes through the same processing as does the protein target that is being assayed, to control for possible losses during these processes [22]. In effect, when properly designed and added at the beginning of the assay, the IRS serves as a normalizer for all sample processing and data acquisition steps – analyte extraction, reduction/alkylation, digestion and MS response. In contrast, most targeted proteomics assays advocate the use of surrogate peptides and their corresponding isotope labeled counterparts as internal reference standards [23], [24], which are added to the sample *after* the initial tryptic digest of the plasma, and can lead to incorrect quantitative results due to incomplete digestion of the targeted protein. The use of isotope-labeled protein standards for quantification (added to the sample prior to processing) has been suggested as a better alternative [25], but they remain expensive and difficult to manufacture. In contrast, readily available protein analogues can also serve as adequate IRS. We have used such analogues in the past, and most of them have been proteins from other species that differ very little in the amino acid sequence [7], [26], [27]. In this work we utilized long arginine 3-IGF1 (LR3-IGF1) as an internal reference standard. LR3-IGF1 differs from native IGF1 in that it has an arginine instead of glutamic acid at position 3 in the amino acid sequence, and it contains extra 13 amino acids at the N-terminus (MFPAMPLLSLFVN), the net result of which is reduced binding of LR3-IGF1 to the IGFBPs. Other possibilities for an IRS include rat IGF1 [13], mouse IGF1, and R3-IGF1 (which will result in identical transition pairs in the MS/MS as those of LR3-IGF1), all of which are readily available as recombinant proteins. We determined empirically that the sequence modifications in LR3-IGF1 did not influence the binding to the IGF1 antibody that was used in the subsequent steps of immunoaffinity purification, which is an important prerequisite.

### Assay Development and Optimization

We opted for an immunoaffinity approach to IGF1 preparation for the subsequent MS detection. Medium- to high-concentration plasma proteins (>1 mg/L) can be detected via LC-MS/MS without enrichment [28], but for most others an immuno-enrichment is more often than not a necessary step. Inter-laboratory evaluation of immunoaffinity enrichment peptide multiple reaction monitoring (MRM) MS assays has demonstrated limits of detection of 1 ng/mL [23]. In our past work we have shown that with the use of appropriate devices for immunoaffinity purification, lowest limits of detection (LLOD) of 10 pg/mL or less are possible [19], [29]. The immunoaffinity capture of IGF1 and LR3-IGF1 was achieved through the use of MSIA-Tips – disposable automation research tips fitted with a small porous microcolumn at the distal end, onto which an IGF1 antibody was immobilized using standard protocols [21]. The optimal amount of antibody was empirically determined to be 1 µg/tip, considering both the performance of the assay (primarily the LLOD) and the cost factor for the antibody. The IGF1 affinity capture and elution protocol were designed with simplicity in mind, containing a minimum number of steps needed for successful capture of the proteins from the sample. When performed on the Versette Automated Liquid Handler, the affinity retrieval and elution of IGF1 and LR3-IGF1 was executed in <45 min, for 96 samples at a time. The subsequent trypsin digestion and LC/MS protocols were adapted from our previous work [19], with few modifications as described in the Methods section. The MS/MS transitions that were monitored for the quantification of IGF1 and LR3-IGF1 are listed in **Table 1**.

**Table 1:** MS/MS transitions for IGF1 and LR3-IGF1 used for the quantification.

IGF1	GPETLC(carboxymethyl)GAELVDALQFVC(carboxymethyl)GDR	
	770.32 (+3)	508.181
		607.249
		754.318
		882.377
		995.461

LR3-IGF1	TLC(carboxymethyl)GAELVDALQFVC(carboxymethyl)GDR	
	675.98 (+3)	347.167
		508.181
		607.249
		754.318
		882.377

### Assay Performance

**Figure 1:**
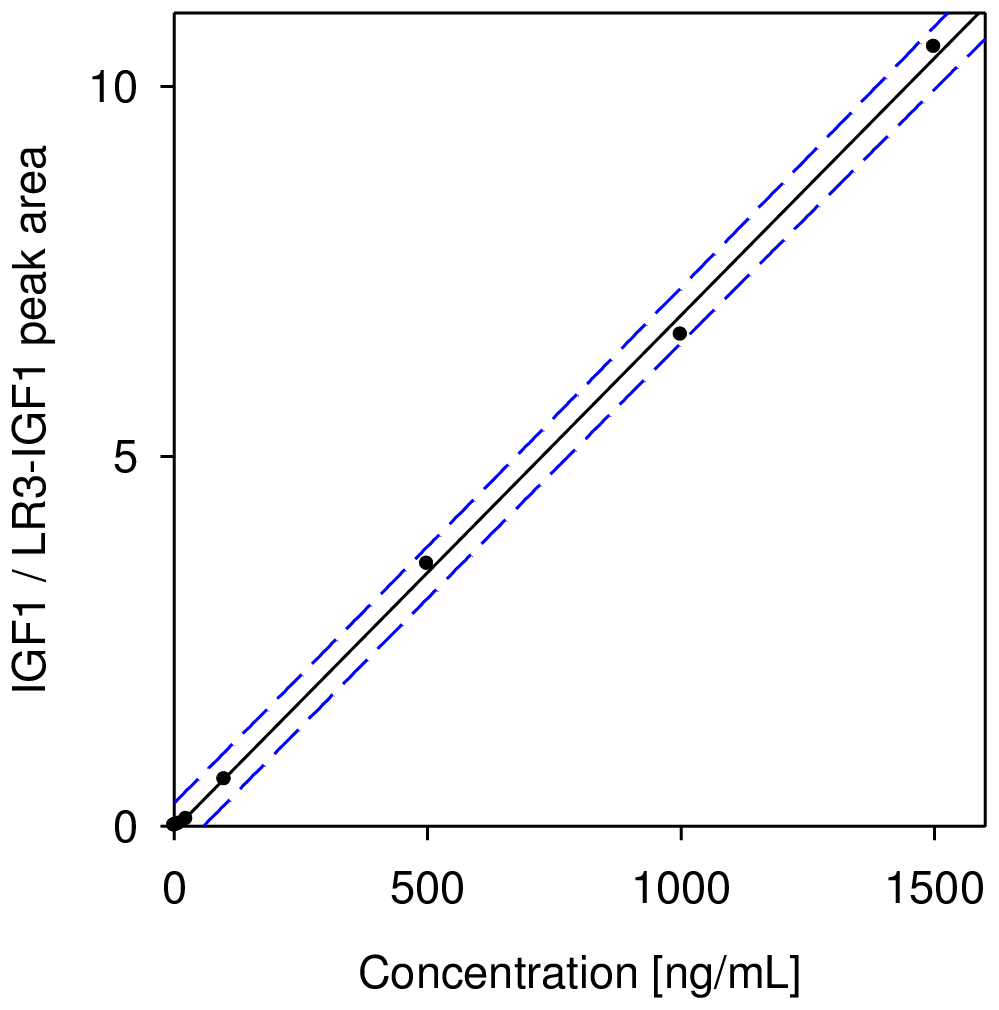
Representative standard curve for the IGF1 SRM-MSIA. Plotted are the peak area ratios of IGF1/LR3-IGF1 over the standards concentrations. Solid line: linear fit with R^2^ = 0.999, SEE = 0.135. Dotted lines: 95% prediction intervals.

Standard curves were generated by plotting the IGF1/LR3-IGF1 peak area ratios vs. the concentration of the IGF1 standards. A typical IGF1 standard curve (**Fig. 1**) covered the range from 1 to 1,500 ng/mL IGF1, with good linearity (R^2^ = 0.999, SEE = 0.135). The lowest limit of detection (LLOD) achieved with the assay was 1 ng/mL (equating to ∼5 femtomoles of IGF1), which was determined as 2 standard deviations above background noise from blank samples. A lower limit of quantifications (LLOQ) of 5 ng/mL was also achieved, as the lowest concentration that was reproducibly quantified. The intra-assay precision (within-run) was determined by analyzing three plasma samples, in triplicates, each with a single standard curve. The inter-assay precision (run-to-run) was determined by analyzing one plasma sample three times, on different days, with separate standard curves each time. The results are shown in **Table 2**, and indicate CVs of less than 10%. To determine the linearity of the assay, plasma samples with known IGF1 concentrations were serially diluted, analyzed with the mass spectrometric immunoassay to determine the IGF1 concentrations, and the results compared to those expected (**Table 3**). Spiking recovery experiments were also performed by spiking plasma samples, with known low IGF1 concentrations, with increasing amounts of recombinant IGF1, followed by analysis with the assay to determine the total IGF1 concentration, and comparison of the results with those expected (**Table 4**).

**Table 2:** Intra-and inter-assay precision.

Intra-assay CVs				Inter-assay CV	
**Sample**	1	2	3		
**STDEVP:**	6.68	9.93	5.88	**STDEVP**	11.1
**MEAN (ng/mL):**	166	149	139	**MEAN (ng/mL)**	151
**CV (%):**	4.03	6.67	4.24	**CV (%)**	7.36

**Table 3:** Assay linearity.

Sample	Dilution	Observed	Expected	Recovery
		ng/mL	ng/mL	O/E%
**1**		306		
	2×	146	153	95.4
	4×	72.7	77.0	94.4
	8×	39.1	38.0	103
**2**		239		
	2×	131	119	110.1
	4×	57.0	60.0	95.0
	8×	34.0	30.0	113

**Table 4:** Spiking recovery.

Sample	Observed	Expected	Recovery
	ng/mL	ng/mL	O/E%
**1**	117		
	229	217	106
	385	317	121
	588	517	114
**2**	107		
	322	294	110
	546	482	113
	757	857	88.3

### Method Comparison

**Figure 2:**
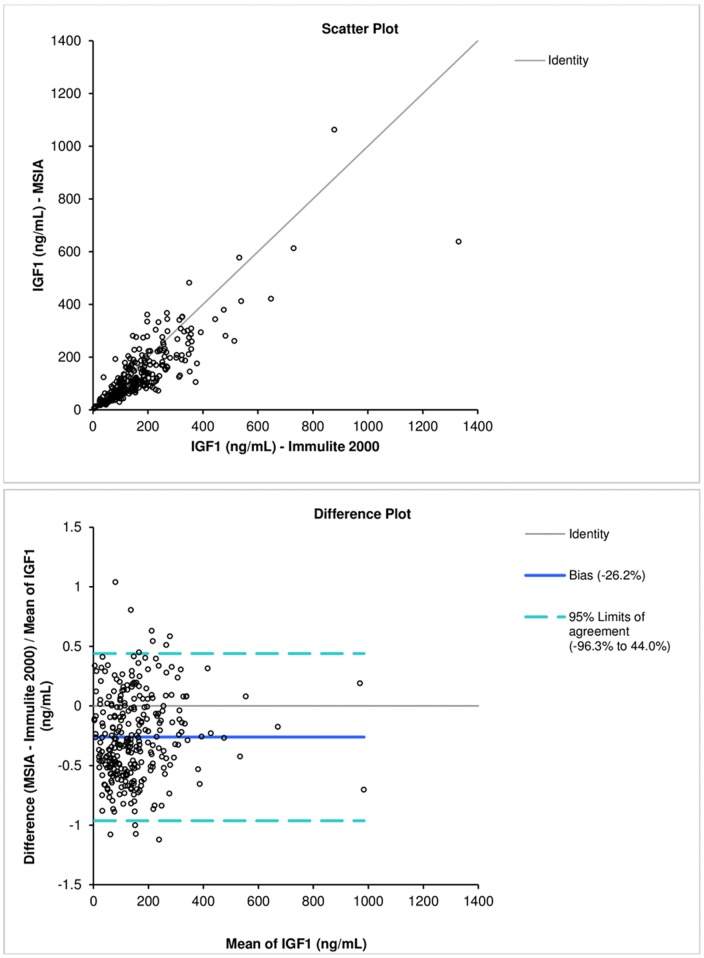
IGF1 methods comparison. a) Scatter plot; b) Difference plot.

The fully-developed, characterized, and validated IGF1 SRM-MSIA assay was then used to analyze a set (n = 289) of human serum samples. The samples were provided by the University Health Network, with their IGF1 levels determined by the Siemens Immulite 2000 Platform [30]. The IGF1 concentrations determined with the SRM-MSIA correlated well with those obtained with the Immulite System, with a Passing Bablock fit [31] of −9.11+0.82x. The Bland-Altman plot [32] shows a slight negative bias of 26% (**Fig. 2**). This is not unusual, and it has been observed in a number of method comparison studies [12], [33]. Further studies might be needed to delineate the source of these deviations, which is particularly important for samples with high IGF1 (e.g. in patients with growth hormone excess - acromegaly; similarly, the effects of increased IGFBP levels on the performance of the assay would need to be further evaluated). The IGF1 SRM-MSIA assay LLOD (1 ng/mL) is lower than the LOD of the Immulite assay (20 ng/mL), and that of a recently published LC-MS/MS assay by Bystrom *et al*. −4 ng/mL [12]. Before the IGF1 SRM-MSIA assay can find clinical application it will need calibrating to the latest IGF-1 international standard (02/254) [34].

## Conclusions

Mass spectrometry-based workflows offer a viable alternative to standard IGF1 immunoassays. While some MS-based methods have been developed for assaying IGF1 from blood spots [35] or urine [15], human plasma remains the most clinically important sample for IGF1 analysis, which requires pre-processing steps in order to contend with IGFBPs. In this work, we have taken the approach of disrupting IGF1/IGFBP complexes *in situ*, as well as streamlined post-processing steps in order to create a highly accurate and reproducible IGF1 SRM MSIA. Critical to the assay is the inclusion of the IRS prior to any pre-processing, extraction, post-processing and analysis steps. Thus, with a single, judiciously chosen IRS, all sources of assay variability can be normalized. Importantly, we have utilized immunoaffinity purification in a *standardized* format to enhance the performance of the assay [36]. While one might argue that immuno-enrichment is not necessary with regards to IGF1, we found it to be necessary for the optimized sample preparation workflow presented in this work (the influence of heterophilic antibodies can be eliminated via the use of blocking tubes [37]). Furthermore, immunoaffinity purification is a prerequisite for detection of most low-abundance proteins, as well as variants of those high- and medium-abundance proteins that are typically found at much lower concentration than their wild-type counterpart. Mass spectrometry, when coupled with immunoaffinity separation, has the necessary sensitivity, automation, and high-throughput capability needed for running hundreds of samples per day. Lastly, in this work we performed trypsin digestion (post-IGF1 capture and elution) only because the triple quadrupole mass spectrometer employed has a better sensitivity at the lower peptide mass range. Others have shown that digestion is not needed for a MS-based IGF1 assay detection [7], [13], and we certainly recognize the fact that without digestion the entire IGF1 assay is much simpler. In that respect we expect to adopt the sample preparation and immunoaffinity steps described in this work to a workflow and assay that employs an advanced MS platform with matched sensitivities to detect intact IGF1. Such assays are certain to find use in the clinical laboratories.
